# Rearrangement of MICU1 multimers for activation of MCU is solely controlled by cytosolic Ca^2+^

**DOI:** 10.1038/srep15602

**Published:** 2015-10-22

**Authors:** Markus Waldeck-Weiermair, Roland Malli, Warisara Parichatikanond, Benjamin Gottschalk, Corina T. Madreiter-Sokolowski, Christiane Klec, Rene Rost, Wolfgang F. Graier

**Affiliations:** 1 Institute of Molecular Biology and Biochemistry, Center of Molecular Medicine, Medical University of Graz, Harrachgasse 21/III, 8010 Graz, Austria

## Abstract

Mitochondrial Ca^2+^ uptake is a vital process that controls distinct cell and organelle functions. Mitochondrial calcium uptake 1 (MICU1) was identified as key regulator of the mitochondrial Ca^2+^ uniporter (MCU) that together with the essential MCU regulator (EMRE) forms the mitochondrial Ca^2+^ channel. However, mechanisms by which MICU1 controls MCU/EMRE activity to tune mitochondrial Ca^2+^ signals remain ambiguous. Here we established a live-cell FRET approach and demonstrate that elevations of cytosolic Ca^2+^ rearranges MICU1 multimers with an EC_50_ of 4.4 μM, resulting in activation of mitochondrial Ca^2+^ uptake. MICU1 rearrangement essentially requires the EF-hand motifs and strictly correlates with the shape of cytosolic Ca^2+^ rises. We further show that rearrangements of MICU1 multimers were independent of matrix Ca^2+^ concentration, mitochondrial membrane potential, and expression levels of MCU and EMRE. Our experiments provide novel details about how MCU/EMRE is regulated by MICU1 and an original approach to investigate MCU/EMRE activation in intact cells.

Mitochondrial Ca^2+^ uptake stimulates mitochondrial dehydrogenases and, thus, oxidative phosphorylation, that, ultimately results in increased ATP formation^1^,^2^. Due to the effective mitochondrial Ca^2+^ buffer capacity local and global Ca^2+^ signals are shaped by mitochondria, thus, these organelles control multiple Ca^2+^-sensitive cell signaling pathways and functions^3^. However, mitochondrial Ca^2+^ overload is associated with an increased generation of reactive oxygen species, cell dysfunctions and the induction of cell death pathways^4^.

While the phenomenon of mitochondrial membrane potential-dependent Ca^2+^ uniport is known since the early 1960ties, the identity of key protagonists that actually accomplish and directly regulate mitochondrial Ca^2+^ uptake remained elusive until very recently. In 2011 a 35-kDa protein, which was initially referred to as CCDC109A and then renamed mitochondrial Ca^2+^ uniporter (MCU) was described to show many if not all of the typical characteristics of a Ca^2+^ uniporter^5^,^6^. It has been suggested that MCU, which possesses two transmembrane domains, forms tetrameric highly specific mitochondrial Ca^2+^ channels. Interestingly, these MCU oligomers are part of larger protein complexes of the inner mitochondrial membrane, indicating the existence of complex mitochondrial Ca^2+^ uptake machineries that include other regulatory subunits^7^. While mitochondrial calcium uniporter regulator 1 (MCUR1)^8^, leucine zipper/EF hand-containing transmembrane-1 (Letm1)^9^,^10^,^11^ and the novel uncoupling proteins 2 and 3 (UCP2/3)^12^,^13^,^14^, which have been shown to contribute to certain modes of mitochondrial Ca^2+^ uptake, have not been found in MCU containing protein complexes^5^, other types of proteins have been identified and characterized as regulatory components of the MCU machinery. These proteins include the dominant-negative pore-forming subunit MCUb^15^, the essential MCU regulator (EMRE)^16^, mitochondrial calcium uptake 1 (MICU1)^17^,^18^ and its isoforms^19^,^20^. MICU1, which was identified some months earlier than MCU, has two classical EF-hand Ca^2+^-binding domains and forms large complexes of app. 480 kDa^17^,^20^. The interaction of MICU1 with MCU is mediated by EMRE^16^,^21^. The two Ca^2+^-binding EF-hand domains of MICU1 can undergo large conformational changes upon binding of Ca^2+^ 22,23 and face the intermembrane space^18^. MICU1 is a gatekeeper of MCU preventing an increase of the mitochondrial Ca^2+^ concentration ([Ca^2+^]_mito_) at low resting Ca^2+^ levels while it is thought to facilitate mitochondrial Ca^2+^ uptake upon high Ca^2+^ concentrations^18^,^24^,^25^. Recently, an elegant study determined the crystal structure of MICU1 indicating that in the absence of Ca^2+^ MICU1 exists as a hexamer, while Ca^2+^ binding to the two EF-hands results in a rearrangement to MICU1 dimers^23^. This study provided the first insights into the molecular mechanism by which MICU1 possibly controls MCU/EMRE activity and suggests that the hexameric MICU1 prevents MCU/EMRE Ca^2+^ channel activity that gets released upon (re-)organization of MICU1 hexamers/multimers^7^. However, whether or not Ca^2+^-dependent rearrangements of MICU1 multimers indeed occur and regulate the activity of MCU in intact cells, and the impact of MCU and EMRE to the structural (re-)organization of MICU1 have not been investigated so far. Hence, the kinetics of the rearrangement of the MICU1 multimers upon cytosolic Ca^2+^ elevation and whether or not the mitochondrial matrix Ca^2+^ concentration or membrane potential (ψ_mito_) have an impact on the structural reorganization of MICU1 remain elusive.

In this study we used a FRET-based live-cell imaging approach to dynamically monitor the kinetics of the structural (re-)organization of MICU1 and to explore its dependence from cytosolic and mitochondrial Ca^2+^ signals as well as ψ_mito_. Simultaneous measurements of cytosolic Ca^2+^ with either mitochondrial Ca^2+^ signals or MICU1 rearrangement were performed to verify whether MICU1 multimers indeed retard MCU activation in intact cells. Finally, we tested whether the Ca^2+^-sensitive rearrangement of MICU1 is dependent of the expression level of MCU and EMRE. Our data provide new insights in the dynamics, regulation, and molecular effect of the structural (re-)organization of MICU1 that adds to our current understanding of the complex molecular mechanisms of MCU activation.

## Results

### Ca^2+^ elevations yield rearrangement of MICU1 multimers in intact cells

**Figure 1:**
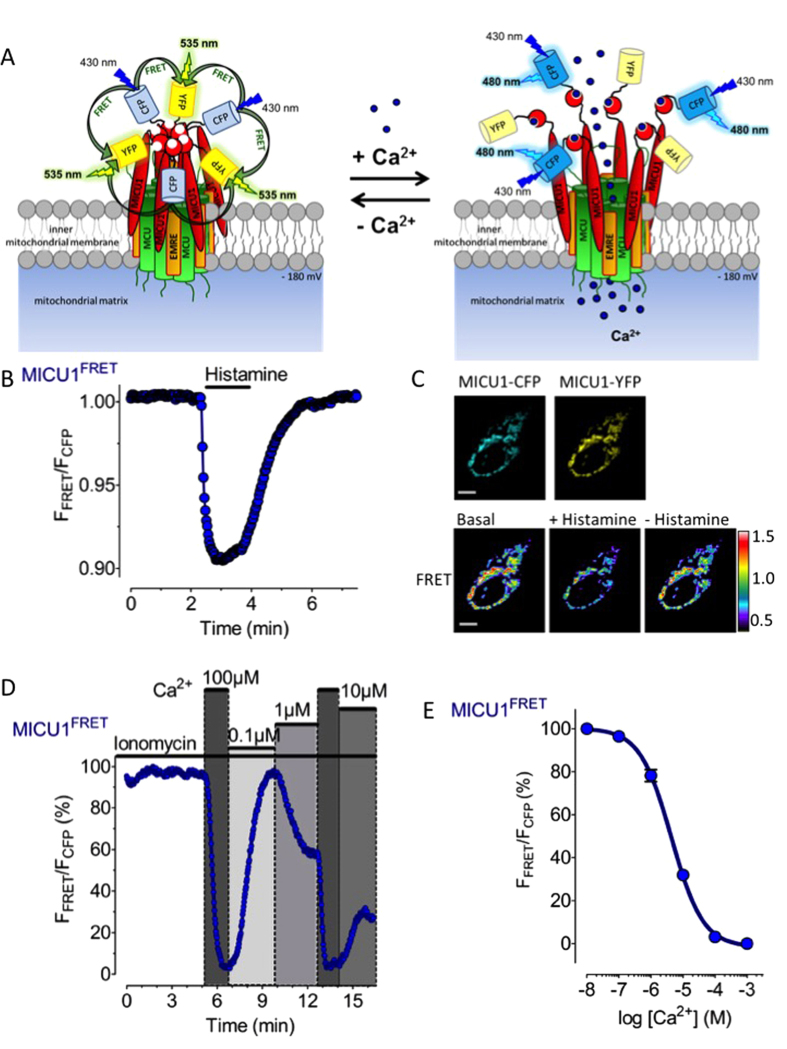
Ca^2+^ dynamically controls rearrangement of MICU1 multimers in intact cells. (**A**) Schematic illustration of the putative Ca^2+^-induced rearrangement of MICU1-CFP and MICU1-YFP hexamers that bind to the MCU/EMRE mitochondrial Ca^2+^ uptake machinery. It is hypothesized that Ca^2+^ binding to the MICU1 EF-hands reduces FRET between the respective MICU1-conjugated FPs. (**B**) Representative curve showing the MICU1 FRET ratio signal of HeLa cells co-expressing MICU1-CFP and MICU1-YFP upon cell treatment with 100 μM histamine in the presence of 2 mM Ca^2+^. (**C**) Representative images to panel b under basal condition, upon stimulation with 100 μM histamine 3 min. after stimulation (scale bar, 10 μm). (**D**) Representative MICU1 FRET ratio signals over time in HeLa cells upon addition and removal of different free Ca^2+^ concentrations in the presence of 3 μM ionomycin. The maximal ∆ MICU1 FRET ratio signal (between 3 mM EGTA and 1000 μM Ca^2+^) was defined as 100%. (**E**) Concentration response curve of the Ca^2+^-induced reduction of the MICU1 FRET ratio signal in HeLa cells that were treated with 3 μM ionomycin and different Ca^2+^ concentrations as shown in panel (**D**); mean ± SEM. (n = 7–9).

In order to dynamically monitor whether and, if so, how intracellular Ca^2+^-mobilization by an inositol 1,4,5-trisphosphate- (IP_3_-)generating agonist affects the arrangement of MICU1 in intact cells, Förster energy transfer (FRET) imaging was applied in cells co-expressing MICU1 fused to either cyan fluorescent protein (MICU1-CFP) or yellow fluorescent protein (MICU1-YFP). Because MICU1 has been shown to assemble in hexamers in the absence of Ca^2+^ and rearranges to dimers upon Ca^2+^ binding^23^, we assumed that under resting conditions the expressed MICU1-CFP and MICU1-YFP chimeras exist as hexamers, thus, facilitating FRET from CFPs to YFPs (Fig. 1A). However, elevation of Ca^2+^ and its subsequent binding to MICU1 should yield disaggregation of MICU1 hexamers and reduce the inter-MICU1 FRET signal. Indeed, cell treatment with the IP_3_-generating agonist histamine^11^ considerably reduced the inter-MICU1 FRET ratio (Fig. 1B,C), while the agonist had no effect on fluorescence of cells expressing MICU1-YFP alone (Supplementary Fig. 1). Upon removal of the agonist the signal was restored (Fig. 1B,C), indicating the reassembly of MICU1 to higher multimers upon the decline of Ca^2+^ levels. In the nominal absence of extracellular Ca^2+^, the histamine-triggered decrease of inter-MICU1 FRET was more transient and slowly developed upon subsequent addition of extracellular Ca^2+^ (Supplementary Fig. 2), thus, highlighting that inter-MICU1 FRET strictly follows cellular Ca^2+^ signals under these conditions (Supplementary Fig. 3). In contrast to the fast intracellular Ca^2+^ mobilization in response to histamine, slow Ca^2+^ mobilization from the endoplasmic reticulum (ER) by the sarcoplasmic/endoplasmic reticulum Ca^2+^ ATPase (SERCA) inhibitor 2,5-di-tert-butylhydroquinone (BHQ)^26^ only slowly and weakly reduced the inter-MICU1 FRET signal (Supplementary Fig. 4). Under this condition mitochondrial Ca^2+^ uptake in response to cell treatment with BHQ was largely increased in cells treated with the 3´UTR siRNA against MICU1 (Supplementary Fig. 5), despite an almost unaffected cytosolic Ca^2+^ elevation (Supplementary Fig. 6). These findings indicate that the slow and weak ER depletion with BHQ yields only in insufficient MICU1-rearrangement, while the inhibitory function of MICU1 multimers on MCU remains under these conditions. However, expression of FP tagged MICU1 completely restored the inhibition of mitochondrial Ca^2+^ uptake at low Ca^2+^ (Supplementary Fig. 5). In contrast, using a MICU1 variant mutated in both canonical EF hands^17^,^23^ was neither able to rescue this siRNA mediated effect of MICU1 (Supplementary Fig. 7) nor showed any MICU1 FRET rearrangement upon stimulation with histamine (Supplementary Fig. 8).

In order to determine the affinity of the MICU1 multimers for Ca^2+^ to evoke their rearrangement *in situ*, the Ca^2+^ ionophore ionomycin was used to clamp different cytosolic Ca^2+^ concentrations (Fig. 1D,E). These experiments revealed a half maximal effective Ca^2+^ concentration (EC_50_) to trigger rearrangement of the MICU1 multimers of 4.4 (3.7–5.2) μM in HeLa cells (Fig. 1E). Our findings are in line with studies demonstrating the existence of Ca^2+^ micro-domains of up to 16 μM in hot spots between the ER and mitochondria in response to an IP_3_-generating agonist^27^. Such Ca^2+^ hot spots would efficiently destruct MICU1 multimers and, hence, activate mitochondrial Ca^2+^ uniport. Our results provide a first demonstration that MICU1 oligomerization is reversibly controlled by high and low [Ca^2+^]_cyto_ in intact cells.

### Ca^2+^-triggered MICU1 de-multimerization occurs prior to mitochondrial Ca^2+^ uptake

**Figure 2:**
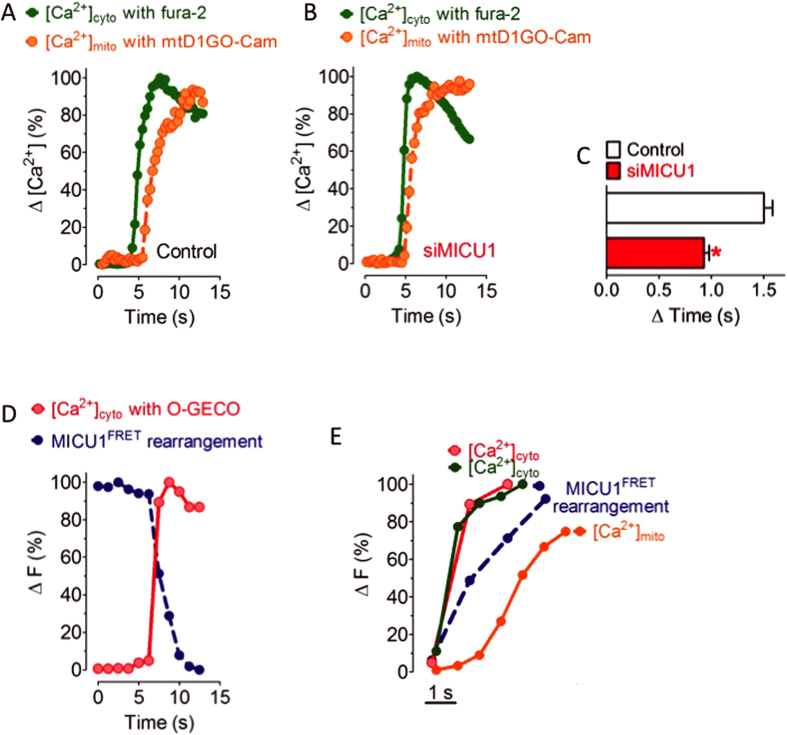
Rearrangement of MICU1 multimers occurs prior to mitochondrial Ca^2+^ uptake. (**A**, **B**) Fura-2/AM loaded cells expressing 4mtD1GO-Cam were used to simultaneously record [Ca^2+^]_cyto_ (green traces) and [Ca^2+^]_mito_ (orange traces) in response to cell treatment with 100 μM histamine in the absence of extracellular Ca^2+^ in control HeLa cells (**A**) and cells reduced of MICU1 (**B**). The respective ∆ ratio values were defined as 100%. (**C**) Bar graph showing ∆ mean time values ± SEM. between the onset of cytosolic and mitochondrial Ca^2+^ signals in response to 100 μM histamine of individual control HeLa cells (white column, n = 7) and cells diminished of MICU1 (red column, n = 9). *P < 0.05 vs. control. (**D**) Representative simultaneous recording of [Ca^2+^]_cyto_ and MICU1 FRET in response to 100 μM histamine under Ca^2+^-free conditions using HeLa cells co-expressing O-GECO, MICU1-CFP and MICU1-YFP. (**E**) Temporal correlation of [Ca^2+^]_cyto_, [Ca^2+^]_mito_ and MICU1 FRET in response to cell treatment with 100 μM histamine in the absence of extracellular Ca^2+^. The two traces for cytosolic Ca^2+^ represent that of fura-2 (green line) and O-GECO (red line) that were measured simultaneously with mtD1GO-Cam (orange line) and MICU1 FRET (blue dotted trace), respectively.

Simultaneous imaging of cytosolic and mitochondrial Ca^2+^ signals in individual single cells using a red-shifted mitochondria targeted cameleon (mtD1GO-Cam) in combination with the near ultra-violet excitable cytosolic Ca^2+^ sensor fura-2 was performed in order to compare the kinetics of cytosolic and mitochondrial Ca^2+^ signals. This approach revealed a lag time (ΔT) of 1.50 ± 0.08 s between the cytosolic Ca^2+^ rise and its transition into the mitochondrial matrix upon intracellular Ca^2+^ mobilization by the IP_3_-generating agonist histamine (Fig. 2A). The molecular mechanism responsible for this temporal shift between rises of [Ca^2+^]_cyto_ and [Ca^2+^]_mito_ is so far unknown. To investigate whether or not the Ca^2+^-dependent de-oligomerization of MICU1 multimers might retard mitochondrial Ca^2+^ signals, identical experiments were performed in cells transfected with siRNA specifically against MICU1. In these MICU1-diminuted cells the coupling between cytosolic and mitochondrial Ca^2+^ signals was greatly improved and the mitochondrial Ca^2+^ signal followed almost instantly the cytosolic Ca^2+^ elevation upon histamine stimulation (ΔT = 0.93 ± 0.05 s) (Fig. 2B,C).

In order to visualize how the temporal pattern of MICU1 de-oligomerization refers to cytosolic Ca^2+^ signals, we imaged dynamic changes of MICU1 FRET simultaneously with cytosolic Ca^2+^ in response to histamine (Fig. 2D). This approach revealed that MICU1 de-oligomerization is only slightly delayed from the histamine-induced increase of [Ca^2+^]_cyto_ and, thus, clearly precedes mitochondrial Ca^2+^ uptake (Fig. 2E). Notably, app. 90% of the maximal histamine-induced increase of [Ca^2+^]_cyto_ trigger ≥ 50% of MICU1 de-oligomerization that appears essential to initiate considerable uptake of Ca^2+^ by mitochondria (Fig. 2D,E). These data dissect the transfer of cytosolic Ca^2+^ into mitochondria into (at least) four sequential steps: (*i*) cytosolic Ca^2+^ elevation and transfer into the intermembrane space, (*ii*) binding of Ca^2+^ to MICU1 oligomers, (*iii*) MICU1 de-oligomerization, and (*IV*) activation of the MCU.

### Rearrangement of MICU1 multimers occurs independently of the mitochondrial membrane potential and matrix Ca^2+^ elevation

**Figure 3:**
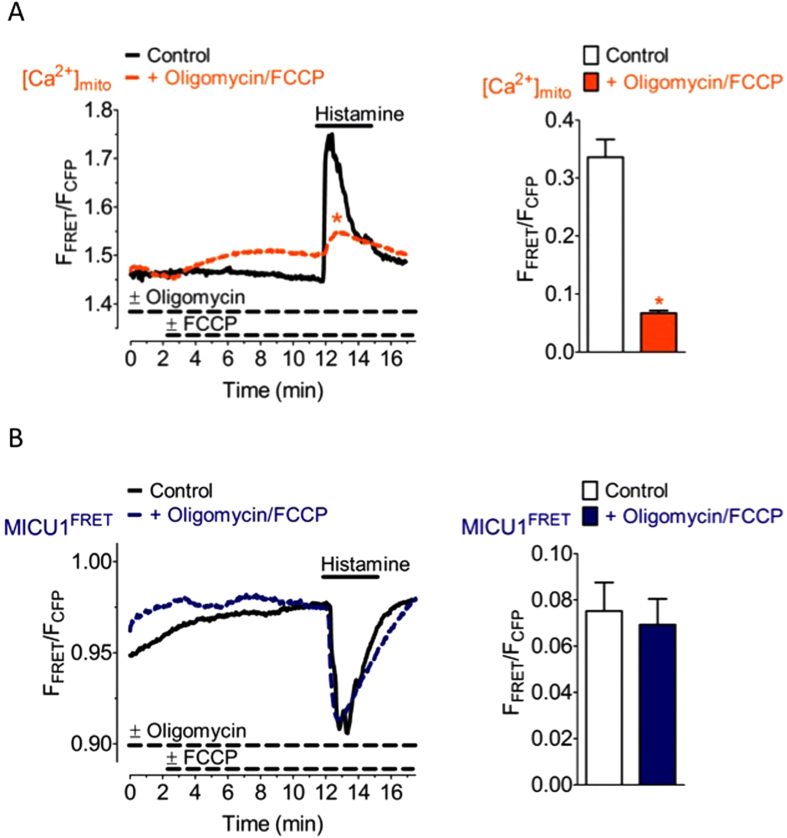
The Ca^2+^-induced rearrangement of MICU1 multimers is independent of Ψ_mito_. (**A**) Average curves reflecting mitochondrial Ca^2+^ signals over time of HeLa cells expressing 4mtD3cpv upon cell treatment with 100 μM histamine in the absence of extracellular Ca^2+^ under control conditions (black curve, n = 10) and in the presence of 2 μM oligomycin and 4 μM FCCP (orange curve, n = 14). Bar graph shows respective maximal ∆ ratio signals; mean ± SEM. *P < 0.05 vs. control. (**B**) Average MICU1 FRET ratio signals of HeLa cells co-expressing MICU1-CFP and MICU1-YFP in response to 100 μM histamine in Ca^2+^-free conditions in the absence of oligomycin and FCCP (control, black curve, n = 3) and the presence of 2 μM oligomycin and 4 μM FCCP (blue curve, n = 6). Bar graph shows respective maximal ∆ MICU1 FRET ratio signals; mean ± SEM.

In order to test if the mitochondrial membrane potential (Ψ_mito_) impacts rearrangement of MICU1 multimers, cells were treated with oligomycin and the uncoupling agent carbonyl cyanide-4-(trifluoromethoxy) phenylhydrazone (FCCP) to efficiently depolarize mitochondria^12^, thus, only very small mitochondrial Ca^2+^ signals in response to histamine were observed (Fig. 3A) approving the loss of Ψ_mito_ under these conditions. However, depolarization of mitochondria did neither affect basal MICU1 FRET nor the histamine-triggered reduction of the inter-MICU1 FRET signal (Fig. 3B). These data indicate that the rearrangement of MICU1 multimers upon intracellular Ca^2+^ mobilization with an IP_3_-generating agonist occurs independently of Ψ_mito_ and the mitochondrial matrix Ca^2+^ elevation.

### MICU1 multimers rearrange irrespective of the expression level of MCU and EMRE

**Figure 4:**
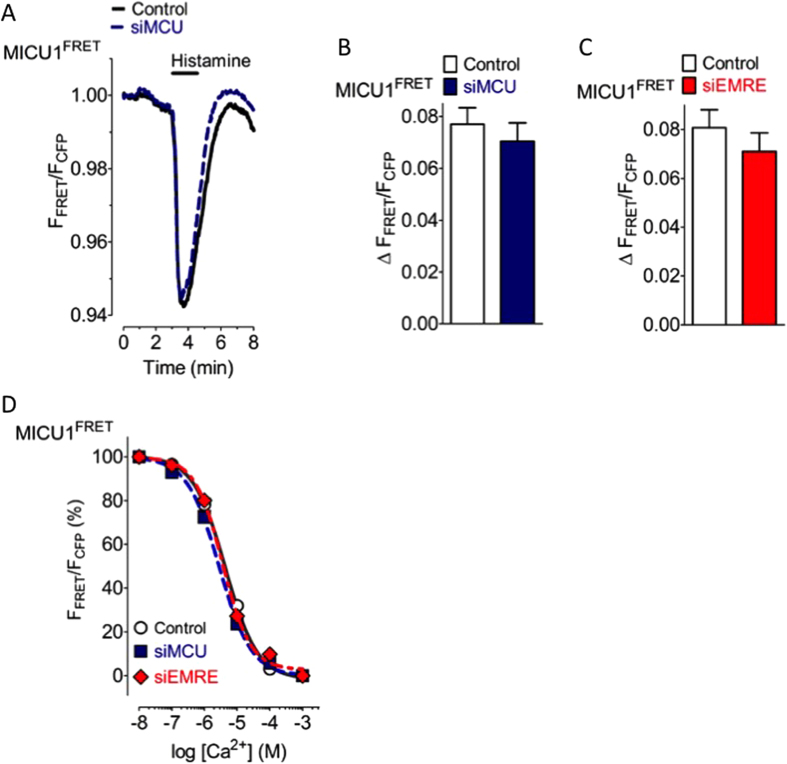
The Ca^2+^-induced rearrangement of MICU1 multimers is independent of the expression level of MCU and EMRE. (**A**) Average curves of MICU1 FRET ratio signals over time in response to 100 μM histamine in Ca^2+^-free solution of control HeLa cells (black curve, n = 23) and cells treated with siRNA against MCU (blue curve, n = 22). (**B**) Bars represent maximal ∆ MICU1 FRET ratio values (mean ± SEM) extracted from curves shown in panel A. (**C**) Bars represent maximal ∆ MICU1 FRET ratio values upon cell treatment with 100 μM histamine in Ca^2+^-free solution of control HeLa cells (white column, n = 19) and cells treated with siRNA against EMRE (red column, n = 12). (**D**) Concentration response curves showing the effects of different Ca^2+^ concentrations on the MICU1 FRET ratio in ionomycin- (3 μM) treated control HeLa cells (black curve, white circles, n = 7–9), cells reduced of MCU (blue dotted curve, blue filled squares, n = 8–10), and cells reduced of EMRE (red dotted curve, red filled rhombs, n = 10).

MICU1 is known to interact with EMRE^16^ that represents the second pore forming protein of the mitochondrial Ca^2+^ complex beside MCU^28^,^29^,^30^, which is regulated by MICU1^19^,^21^,^25^. To investigate the importance of EMRE and MCU for the Ca^2+^-triggered rearrangement of MICU1 multimers, expression of either EMRE or MCU were diminished by respective verified siRNAs (Supplementary Fig. 9). Diminution of the expression of either MCU or EMRE strongly reduced mitochondrial Ca^2+^ uptake (Supplementary Fig. 10) while no effect on cytosolic Ca^2+^ signaling was observed (Supplementary Fig. 11). However, the rearrangement of the MICU1 multimers upon stimulation with histamine was neither affected by knock-down of MCU (Fig. 4A,B) nor EMRE (Fig. 4C). In line with these findings the EC_50_ of Ca^2+^ to reduce the inter-MICU1 FRET signal remained unaffected in cells diminished of either MCU or EMRE (Fig. 4D).

## Discussion

The present study describes the dynamics of MICU1 (re-)organization in response to cytosolic Ca^2+^ elevations in intact cells. Using the FRET technology we could correlate the Ca^2+^ induced rearrangement of MICU1 multimers with the activation of mitochondrial Ca^2+^ uniport and examined the impact of mitochondrial Ca^2+^, Ψ_mito_ and the expression levels of MCU and EMRE for MICU1 (re-)organization. Our data highlight that an elevation of cytosolic free Ca^2+^ rearranges MICU1 multimers to smaller complexes in intact cells. These findings are consistent with a recent report showing that recombinant Ca^2+^-free MICU1 exists as hexamer and rearranges in the presence of Ca^2+^ to dimers^7^,^23^. Hence, our data that point to the existence of large MICU1 complexes that suppresses mitochondrial Ca^2+^ uptake at low cytosolic Ca^2+^ is in line with previous reports about the inactivity of MCU under resting conditions^8^,^18^.

Our approach of live-cell monitoring the inter-MICU1 FRET allows the visualization of MICU1 (re-)organization within the intact cell in life-time. Notably, calculation of the changes in MICU1 FRET probability in the transition from hexamers to dimers revealed a theoretically achievable reduction of FRET by app. 52% (Supplementary Information, Supplementary Fig. 12). However, the actual measured changes in FRET upon MICU1 rearrangement triggered by histamine was app. 7%. The discrepancy between the theoretical FRET changes upon the rearrangement of hexamers to independent dimers and the actual measured one might be due to basically two reasons: *first*, the high expression of FP tagged MICU1 proteins might result in lower oligomerization states, and *second*, upon histamine-induced intracellular Ca^2+^ release, MICU1 de-oligomerization most likely occurs predominantly within the junctions between the ER and mitochondria where high Ca^2+^ gradients are developed^27^, thus, only a small portion of MICU1 hexamers actually undergoes rearrangement under this conditions. This assumption is further supported by the app. 15% FRET reduction achieved in the assessment of the concentration response curve of MICU1 FRET dynamic to Ca^2+^.

Our approach that allowed correlation of the kinetics of MICU1 (re-)organization with cellular Ca^2+^ dynamics data revealed that the rearrangement of MICU1 multimers by cytosolic Ca^2+^ strictly correlates with the cytosolic Ca^2+^ concentration and is rapidly reversible. The *in situ* calibration indicates that the rearrangement of MICU1 senses Ca^2+^ changes in the range between the low basal Ca^2+^ levels of 100 nM up to 100 μM. With an EC_50_ of around 4 μM, the Ca^2+^ sensitivity of MICU1 rearrangement lays exactly in the range of Ca^2+^ hot spots (3.78 and 16.4 μM) that have been measured at the outer mitochondrial membrane between the junction of mitochondria and the ER^27^. In line with this report, the IP_3_-mediated intracellular Ca^2+^ mobilization almost instantly yielded MICU1 rearrangement, while Ca^2+^entry via the store-operated Ca^2+^ entry pathway^31^,^32^ triggered only a slow and moderate re-organization of MICU1 multimers. These differences between the MICU1 rearrangement upon intracellular Ca^2+^ release and entering Ca^2+^ are consistent with previous reports that described a rather slow on-kinetics of entering Ca^2+^ at the mitochondrial surface without the formation of Ca^2+^ hot spots and slow mitochondrial Ca^2+^ accumulation^14^,^27^,^33^.

Similar to entering Ca^2+^, the inhibition of SERCA by BHQ yielded only slow and small rearrangement of MICU1 multimers and, thus, explains the marginal effect of SERCA inhibition on mitochondrial Ca^2+^ uptake. Since the diminution of MICU1 expression substantially gains mitochondrial Ca^2+^ uptake in response to intracellular Ca^2+^ release by SERCA inhibition, the prominent role of MICU1 as a negative regulator of mitochondrial Ca^2+^ uniport is clearly demonstrated. This assumption is further supported by our experiments in which cytosolic and mitochondrial free Ca^2+^ were simultaneously measured and the lag time of app. 1.5 s between cytosolic Ca^2+^ elevation and the mitochondrial Ca^2+^ uptake was strongly reduced in cells with diminished expression of MICU1. Hence, the correlation between cytosolic Ca^2+^ elevation, the rearrangement of MICU1 multimers and mitochondrial Ca^2+^ signals, revealed that MICU1 reorganization temporally occurs in between the Ca^2+^ rise within the two compartments. Notably, significant mitochondrial Ca^2+^ uptake appears to occur at app. 50% rearrangement of MICU1 multimers. These data demonstrate that for activation of the MCU/EMRE complex to achieve mitochondrial Ca^2+^ influx, app. 50% of the MICU1 multimers have to be rearranged. Although these findings might be due to the overexpression of MICU1 in our model, the MICU1-dependent lack of mitochondrial Ca^2+^ uptake in response to SERCA inhibition supports this assumption.

Mitochondrial Ca^2+^ uptake strongly depends on Ψ_mito_ that establishes the driving force for Ca^2+^ uniport into the organelle. However, whether or not Ψ_mito_ also impacts on MICU1 organization has not been investigated so far. Our data with completely depolarized mitochondria revealed an unchanged MICU1 dynamics despite a greatly reduced mitochondrial Ca^2+^ uptake. These data demonstrate that neither Ψ_mito_ nor matrix Ca^2+^ are involved in the rearrangement of MICU1 complexes and confirms cytosolic Ca^2+^ as possible the sole regulator of MICU1 (re-)organization^23^.

Considering the interaction of MICU1 with EMRE and MCU^7^,^16^,^18^,^19^,^23^,^25^,^29^, the importance of these two pore-forming proteins^16^,^28^,^29^ for the structural organization of MICU1 was evaluated. Although the siRNA-mediated diminution of either of these proteins resulted in strongly reduced mitochondrial Ca^2+^ uptake, the Ca^2+^-triggered rearrangement of MICU1 multimers, their arrangement upon the reduction of cytosolic Ca^2+^ to basal levels, and the sensitivity to cytosolic Ca^2+^ remained unaffected by the reduction of MCU or EMRE. These data demonstrate that the structural (re-)organization of MICU1 upon elevation of cytosolic free Ca^2+^ does not involve MCU or EMRE indicating that the Ca^2+^-induced rearrangement of MICU1 multimers is a robust process that does most likely not require other interaction partners.

Despite the topology of MICU1 is still under debate^18^,^21^,^25^,^30^, our data that, *first*, MICU1 FRET rearrangement follows cytosolic but not matrix mitochondrial Ca^2+^, *second*, MICU1 knockdown results in a faster mitochondrial Ca^2+^ uptake, *third*, rearrangement of MICU1 FRET precedes mitochondrial Ca^2+^ uptake, *forth*, MICU1 FRET is independent from Ψ_mito_, and *fifth*, neither knockdown of MCU nor of EMRE influences MICU1 FRET rearrangement indicate that MICU1 anchors with its N-terminus in the IMM while the core protein is oriented towards intermembrane space and not to the matrix.

Our results provide new mechanistic insights in the regulation of mitochondrial Ca^2+^ uptake. For the first time, the kinetics and adjustments of one of the most important molecular gatekeeper of mitochondrial Ca^2+^ uptake was visualized in intact cells. Our data revealed cytosolic Ca^2+^ as the most prominent regulator of the structural organization of MICU1, which in the form of a homo-multimere potently inhibits the MCU/EMRE mitochondrial Ca^2+^ channel complex. Moreover, neither Ψ_mito_ nor matrix Ca^2+^, nor MCU or EMRE were found to affect the Ca^2+^-controlled (dis)assembly of MICU1 multimers. Finally, our data provide important details for a better understanding of the molecular regulation of an intricate mitochondrial Ca^2+^ uptake machinery.

## Methods

### Chemicals and Buffers

All reagents and chemicals for buffers and solutions were obtained from Carl Roth (Karlsruhe, Germany). Fura-2/AM was obtained from Teflabs (Texas Fluorescence Labs Inc., Austin, Texas, USA). BHQ and histamine were purchased from Sigma-Aldrich (Vienna, Austria). FCCP and oligomycin were from Abcam Biochemicals (Cambridge, UK). Prior to experiments HeLa cells were stored and loaded with fura-2/AM in a buffer composed of (in mM): 2 CaCl_2_ 135 NaCl, 5 KCl, 1 MgCl_2_, 1 HEPES, 2.6 NaHCO_3_, 0.44 KH_2_PO_4_, 0.34 Na_2_HPO_4_, 10 D-glucose, 0.1% vitamins, 0.2% essential amino acids and 1% penicillin/streptomycin pH 7.4. For Ca^2+^ measurements HeLa cells were perfused in a HEPES buffered solution containing (in mM): 2 CaCl_2_, 140 NaCl, 5 KCl, 1 MgCl_2_, 1 HEPES and 10 D-Glucose pH 7.4. To achieve a nominal Ca^2+^ free environment either 1 mM EGTA was added instead of 2 mM CaCl_2_. For Kd determination of MICU1 FRET we used the same buffer containing 3 mM EGTA supplemented with 3–10 μM ionomycin (Abcam Biochemicals, Cambridge, UK) and CaCl_2_ was added according to the CaBuff software (G. Droogmans, Fysiologie, Leuven) to obtain buffer solutions of 0.1, 1, 10, 100 and 1000 μM free Ca^2+^ concentrations.

### Cell culture and transfection

HeLa cells at passage >50 were cultured on glass cover slips (Ø = 30 mm) using DMEM (Sigma-Aldrich, Vienna, Austria) containing 10% FCS (PAA, Pasching, Austria), penicillin (100 U/ml) and streptomycin (100 U/ml) in a humidified incubator (37 °C, 5% CO_2_/95% air). 2–3 days prior experiments HeLa cells were transfected in DMEM (without FCS and antibiotics) with plasmids (1–4 μg/ml total) and/or siRNA (100 nM) using 4 μg/ml TransFast^TM^ transfection reagent (Promega, Madison, WI, USA).

### Plasmids and siRNAs

For engineering MICU1-CFP and MICU1-YFP the coding sequence (cds) of human MICU1 (hMICU1, NM_006077.3) without a stop codon was amplified from a HeLa cDNA by PCR using primers (Invitrogen, Vienna, Austria): forward: 5′-ACGGATCCACCATGTTTCGTCTGAACTCAC-3′ and reverse: 5′-ACGAATTCCTGTTTGGGTAAAGCGAAGTCC-3′. The PCR fragment was cloned in a pcDNA3.1 (+) vector via BamHI and EcoRI sites. The cds of either enhanced cyan fluorescent protein (CFP) or citrine (YFP) were amplified with the same primers: forward: 5′-AAGAATTCATGGTGAGCAAGGGCGAGGAG-3′ and reverse: 5′-CCTCTAGAACTTGTACAGCTCGTCCATGC-3′ and each C-terminally fused to hMICU1 using the EcoRI and XbaI sites. EF hand mutated MICU1 and O-GECO1 constructs were purchased from Addgene (Cambridge, MA, USA). The EF hand mutated MICU1 (EFmut) was amplified using the same primers as described above and C-terminally fused with either ECFP or Citrine like the wild-type MICU1. A mitochondrial targeted mtO-GECO1 was generated upon PCR amplification of O-GECO1 using the forward primer 5′-AACTCGAGTATGGTCGACTCATCACGTCG-3′ and a reverse primer 5′-GCAAGCTTTTACTTCGCTGTCATCAT-3′ and the PCR fragment was N-terminally fused with the mitochondrial targeting sequence (4mt) via XhoI and HindIII restriction sites in a pcDNA3.1(–) vector. 4mtD1GO-Cam was constructed as previously described^34^ and 4mtD3cpV (Mt-cameleon-pcDNA3) was a gift from Prof. Dr. Roger Tsien. For silencing hMCU (NM_138357.2), hEMRE (NM_033318.4) or hMICU1 we used siRNAs from Microsynth (Balgach, Switzerland) with following sequences (sense strands, 5′-3′): hMCU-si1 (GCCAGAGACAGACAAUACU), hMCU-si2 (GGAAAGGGAGCUUAUUGAA); hEMRE-si (GAACUUUGCUGCUCUACUU); hMICU1-si1 (GCAGCUCAAGAAGCACUUCAA), hMICU1-si2 (GCAAUGGCGAACUGAGCAAUA) or 3′UTR-siMICU1 (AGAAGUCUGUGAUGAUAAA, binds in the non-coding 3′ terminal untranslated region of MICU1); and a scrambled negative control-si (UUCUCCGAACGUGUCACGU).

### Quantitative reverse transcription PCR

To determine hEMRE and hMCU silencing HeLa cells were transiently transfected with the respective siRNA(s). The PEQLAB total RNA isolation kit (PEQLAB Biotechnologie GmBH, Erlangen, Germany) was used for total RNA isolation and 1 μg of each RNA sample were reverse transcribed with a cDNA synthesis kit (Applied Biosystems, USA). The efficiency of siRNA mediated knockdown was validated by real time PCR on a LightCycler 480 (Roche Diagnostics, Vienna, Austria). Target genes and human GAPDH (no. QT01192646, QuantiTect® Primer Assay, Qiagen, Hilden, Germany) as housekeeping gene were amplified within the respective cDNA samples using the GoTaq® qPCR Master Mix (Promega) and specific real time primer pairs (Invitrogen): hMCU forward: 5′-TTCCTGGCAGAATTTGGGAG-3′, hMCU reverse: 5′-AGAGATAGGCTTGAGTGTGAAC-3′; hEMRE forward: 5′-TCGCTGGCTAGTATTGGCAC-3′, hEMRE reverse: 5′-GGAGAAGGCCGAAGGACATT-3′. Relative expression of the hEMRE and hMCU were normalized to GAPDH expression and data were analyzed by the REST software (Qiagen, Hilden, Germany).

### Fluorescence microscopy

Ca^2+^ imaging and dynamic FRET measurements between MICU1-CFP and MICU1-YFP were performed on an inverted microscope (Axio Observer.A1, Zeiss, Göttingen, Germany) equipped with a polychromator illumination system (VisiChrome, Visitron Systems, Puchheim, Germany) and a thermoelectric-cooled CCD camera (Photometrics CoolSNAP HQ, Visitron Systems). Cells were imaged with a 40× oil-immersion objective (Zeiss). Fura-2 and the 4mtD1GO-Cam were alternately excited at 340 nm or 380 nm and at 477 nm, respectively, with an ultra-fast switching monochromator, the Polychrome V (Till Photonics), equipped with an excitation filter (E500spuv) and a dichroic filter (495dcxru, Chroma Technology Corp., VT). Emitted light was simultaneously collected at 510 nm (Fura-2 and GFP of GO-Cam) and at 600 nm (FRET-channel of GO-Cam) using a single beam splitter design (Dichrotome, Till Photonics) that was equipped with a dual band emission filter (59004m ET Fitc/Tritc Dual Emitter) and a second dichroic filter (560dcxr, Chroma Technology Corp.). Images were recorded with a CCD camera (AVT Stringray F145B, Till Photonics). The digital imaging system was controlled by the live-acquisition software v2.0.0.12 (Till Photonics), as described previously. For the dynamic CFP-YFP FRET measurements transfected cells were excited at 440 ± 10 nm (440AF21, Omega Optical, Brattleboro, VT), and emission was recorded at 480 and 535 nm using emission filters (480AF30 and 535AF26, Omega Optical) mounted on a Ludl filterwheel. Results of FRET measurements are shown as the ratio of F_FRET_/F_CFP_ or as (F_FRET_/F_CFP_)/R_0_ (where R_0_ is the basal ratio), to correct for photobleaching and/or photochromism, as described previously^34^. Dual recordings of MICU1 FRET and [Ca^2+^]_cyto_ was performed in HeLa cells co-transfected with MICU1-CFP, MICU1-YFP and O-GECO1 or mtO-GECO1, respectively. MICU1-CFP and (mt)O-GECO1 were alternately illuminated at 430 nm and 575 nm, respectively, in single individual cells. Emitted light was collected at 535 nm for MICU1 FRET and 610 nm for (mt)O-GECO1 using the XF56 filter set of Omega Optical.

### Statistics

All experiments were repeated at least three times on two different days. Data from multiple experiments were quantified to determine absolute or percent changes, expressed as mean ± SEM where n reflects the actual number of repeats. Statistical analyses were performed with unpaired Student’s t-test. When not normally distributed, a Mann-Whitney *U* test was applied. Differences in means among multiple data sets were analyzed by using one-way analysis of variance (ANOVA) and subsequent Bonferroni *post hoc* test and two-tailed Student’s t-test. P < 0.05 was considered significant. Data were analyzed either with MS Excel 2011 or with Graphpad Prism version 5.0.

## Additional Information

**How to cite this article**: Waldeck-Weiermair, M. *et al.* Rearrangement of MICU1 multimers for activation of MCU is solely controlled by cytosolic Ca^2+^. *Sci. Rep.* **5**, 15602; doi: 10.1038/srep15602 (2015).

## Supplementary Material

Supplementary Information

## References

[b1] Szabadkai G., Pitter J. & Spät A. Cytoplasmic Ca^2+^ at low submicromolar concentration stimulates mitochondrial metabolism in rat luteal cells. Pflugers Arch 441, 678–685 (2001). 11294250 10.1007/s004240000466

[b2] Malli R. & Graier W. F. Mitochondrial Ca^2+^ channels: Great unknowns with important functions. FEBS Lett 584, 1942–1947 (2010). 20074570 10.1016/j.febslet.2010.01.010 PMC4060513

[b3] Szabadkai G. & Duchen M. R. Mitochondria: the hub of cellular Ca^2+^ signaling. Physiology (Bethesda) 23, 84–94 (2008). 18400691 10.1152/physiol.00046.2007

[b4] Duchen M. R., Verkhratsky A. & Muallem S. Mitochondria and calcium in health and disease. Cell Calcium 44, 1–5 (2008). 18378306 10.1016/j.ceca.2008.02.001

[b5] Baughman J. M. *et al.* Integrative genomics identifies MCU as an essential component of the mitochondrial calcium uniporter. Nature 476, 341–345 (2011). 21685886 10.1038/nature10234 PMC3486726

[b6] De Stefani D., Raffaello A., Teardo E., Szabò I. & Rizzuto R. A forty-kilodalton protein of the inner membrane is the mitochondrial calcium uniporter. Nature 476, 336–340 (2011). 21685888 10.1038/nature10230 PMC4141877

[b7] Wang L., Yang X. & Shen Y. Molecular mechanism of mitochondrial calcium uptake. Cell Mol. Life Sci. 72, 1489–1498 (2015). 25548802 10.1007/s00018-014-1810-1 PMC11113575

[b8] Mallilankaraman K. *et al.* MCUR1 is an essential component of mitochondrial Ca^2+^ uptake that regulates cellular metabolism. Nat. Cell Biol. 14, 1336–1343 (2012). 23178883 10.1038/ncb2622 PMC3511605

[b9] Jiang D., Zhao L. & Clapham D. E. Genome-Wide RNAi Screen Identifies Letm1 as a Mitochondrial Ca^2+^/H^+^ Antiporter. Science 326, 144–147 (2009). 19797662 10.1126/science.1175145 PMC4067766

[b10] Waldeck-Weiermair M. *et al.* The leucine zipper EF hand-containing transmembrane protein 1 (LETM1) and uncoupling proteins- 2 and 3 (UCP2/3) contribute to two distinct mitochondrial Ca^2+^ uptake pathways. J. Biol. Chem. 286, 28444–28455 (2011). 21613221 10.1074/jbc.M111.244517 PMC3151087

[b11] Waldeck-Weiermair M. *et al.* Molecularly distinct routes of mitochondrial Ca^2+^ Uptake are activated depending on the activity of the sarco/endoplasmic reticulum Ca^2+^ ATPase (SERCA). J. Biol. Chem. 288, 15367–15379 (2013). 23592775 10.1074/jbc.M113.462259 PMC3663555

[b12] Trenker M., Malli R., Fertschai I., Levak-Frank S. & Graier W. F. Uncoupling proteins 2 and 3 are fundamental for mitochondrial Ca^2+^ uniport. Nat. Cell Biol. 9, 445–452 (2007). 17351641 10.1038/ncb1556 PMC4060164

[b13] Trenker M., Fertschai I., Malli R. & Graier W. F. UCP2/3 — likely to be fundamental for mitochondrial Ca^2+^ uniport. Nat. Cell Biol. 10, 1237–1240 (2008). 10.1038/ncb1556 PMC4060164 17351641

[b14] Waldeck-Weiermair M. *et al.* The contribution of UCP2 and UCP3 to mitochondrial Ca^2+^ uptake is differentially determined by the source of supplied Ca^2+^. Cell Calcium 47, 433–440 (2010). 20403634 10.1016/j.ceca.2010.03.004

[b15] Raffaello A. *et al.* The mitochondrial calcium uniporter is a multimer that can include a dominant-negative pore-forming subunit. EMBO J. 32, 2362–2376 (2013). 23900286 10.1038/emboj.2013.157 PMC3771344

[b16] Sancak Y. *et al.* EMRE is an essential component of the mitochondrial calcium uniporter complex. Science 342, 1379–1382 (2013). 24231807 10.1126/science.1242993 PMC4091629

[b17] Perocchi F. *et al.* MICU1 encodes a mitochondrial EF hand protein required for Ca^2+^ uptake. Nature 467, 291–296 (2010). 20693986 10.1038/nature09358 PMC2977980

[b18] Csordás G. *et al.* MICU1 controls both the threshold and cooperrative activation of the mitochondrial Ca^2+^ Uniporter. Cell Metab. 17, 976–987 (2013). 23747253 10.1016/j.cmet.2013.04.020 PMC3722067

[b19] Patron M. *et al.* MICU1 and MICU2 finely tune the mitochondrial Ca^2+^ uniporter by exerting opposite effects on MCU activity. Mol. Cell. 53, 726–737 (2014). 24560927 10.1016/j.molcel.2014.01.013 PMC3988891

[b20] Plovanich M. *et al.* MICU2, a paralog of MICU1, resides within the mitochondrial uniporter complex to regulate calcium handling. PLoS ONE 8, e55785 (2013). 23409044 10.1371/journal.pone.0055785 PMC3567112

[b21] Hoffman N. E. *et al.* MICU1 motifs define mitochondrial calcium uniporter binding and activity. Cell Reports 5, 1576–1588 (2013). 24332854 10.1016/j.celrep.2013.11.026 PMC3919628

[b22] Gifford J. L., Walsh M. P. & Vogel H. J. Structures and metal-ion-binding properties of the Ca^2+^-binding helix-loop-helix EF-hand motifs. Biochem J 405, 199–221 (2007). 17590154 10.1042/BJ20070255

[b23] Wang L. *et al.* Structural and mechanistic insights into MICU1 regulation of mitochondrial calcium uptake. EMBO J. 33, 594–604 (2014). 24514027 10.1002/embj.201386523 PMC3989653

[b24] Mallilankaraman K. *et al.* MICU1 is an essential gatekeeper for MCU-mediated mitochondrial Ca^2+^ uptake that regulates cell survival. Cell 151, 630–644 (2012). 23101630 10.1016/j.cell.2012.10.011 PMC3486697

[b25] Foskett K. J. & Madesh M. Regulation of the mitochondrial Ca^2+^ uniporter by MICU1 and MICU2. Biochem. Biophys. Res. Commun. 449, 377–383 (2014). 24792178 10.1016/j.bbrc.2014.04.146 PMC5371403

[b26] Osibow K., Malli R., Kostner G. M. & Graier W. F. A new type of non-Ca^2+^-buffering Apo(a)-based fluorescent indicator for intraluminal Ca^2+^ in the endoplasmic reticulum. J. Biol. Chem. 281, 5017–5025 (2006). 16368693 10.1074/jbc.M508583200 PMC4845882

[b27] Giacomello M. *et al.* Ca^2+^ hot spots on the mitochondrial surface are generated by Ca^2+^ mobilization from stores, but not by activation of store-operated Ca^2+^ channels. Mol. Cell 38, 280–290 (2010). 20417605 10.1016/j.molcel.2010.04.003

[b28] Foskett K. J. & Philipson B. The mitochondrial Ca^2+^ uniporter complex. J. Mol. Cell. Cardiol. 78, 3–8 (2015). 25463276 10.1016/j.yjmcc.2014.11.015 PMC4307384

[b29] De Stefani D. & Rizzuto R. Molecular control of mitochondrial calcium uptake. Biochem. Biophys. Res. Commun. 449, 373–376 (2014). 24792182 10.1016/j.bbrc.2014.04.142

[b30] Kamer K. J., Sancak Y. & Mootha V. K. The uniporter: From newly identified parts to function. Biochem. Biophys. Res. Commun. 449, 370–372 (2014). 24814702 10.1016/j.bbrc.2014.04.143

[b31] Putney J. W. J. A model for receptor-regulated calcium entry. Cell Calcium 7, 1–12 (1986). 2420465 10.1016/0143-4160(86)90026-6

[b32] Parekh A. B. Store-operated Ca^2+^ entry: dynamic interplay between endoplasmic reticulum, mitochondria and plasma membrane. J. Physiol. 547, 333–348 (2003). 12576497 10.1113/jphysiol.2002.034140 PMC2342659

[b33] Waldeck-Weiermair M. *et al.* Uncoupling protein 3 adjusts mitochondrial Ca^2+^ uptake to high and low Ca^2+^ signals. Cell Calcium 48, 288–301 (2010). 21047682 10.1016/j.ceca.2010.10.004 PMC2998676

[b34] Waldeck-Weiermair M. *et al.* Spatiotemporal correlations between cytosolic and mitochondrial Ca^2+^ signals using a novel red-shifted mitochondrial targeted cameleon. PLoS ONE 7, e45917 (2012). 23029314 10.1371/journal.pone.0045917 PMC3448721

