# Supplementary material for: Rearrangement of MICU1 multimers for activation of MCU is solely controlled by cytosolic Ca2+
Source: Sci Rep. 2015 Oct 22;5:15602. doi: 10.1038/srep15602 (PMC4615007; doi:10.1038/srep15602)
Supplement: Supplementary Information [file srep15602-s1.pdf]

SUPPLEMENTARY FIGURES & METHOD

to

Rearrangement of MICU1 multimers for activation of MCU is solely controlled by cytosolic Ca^{2+}

Markus Waldeck-Weiermair[#], Roland Malli[#], Warisara Parichatikanond[#], Benjamin Gottschalk, Corina T. Madreiter-Sokolowski, Christiane Klec, Rene Rost, and Wolfgang F. Graier*

[#]equally contributing authors

Institute of Molecular Biology and Biochemistry, Center of Molecular Medicine, Medical University of Graz, Harrachgasse 21/III, 8010 Graz, Austria.

Running title: Visualization of MICU1 rearrangement

Keywords: FRET, imaging, MCU, MICU1, mitochondrial Ca^{2+} uptake, EMRE

***Author of Correspondence:**
Wolfgang F. Graier, Univ. Prof. Dr.
Institute of Molecular Biology and Biochemistry
Research Unit for Molecular and Cellular Physiology, Center of Molecular Medicine
Medical University of Graz
Harrachgasse 21/III,
8010 Graz, Austria
Phone: +43 316 380 7560
Fax: +43 316 380 9615
eMail: wolfgang.graier@medunigraz.at

SUPPLEMENTARY FIGURE 1:

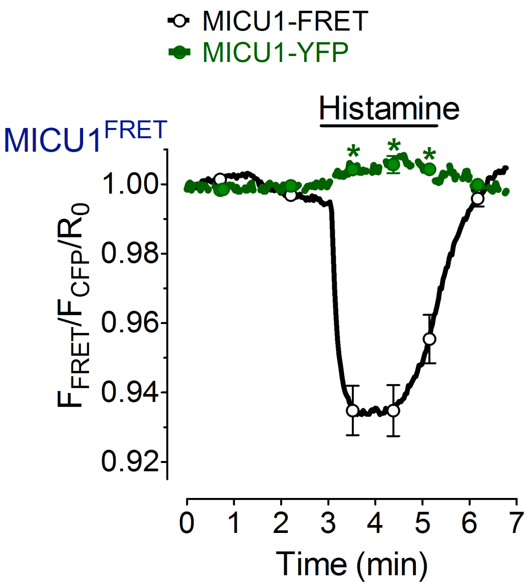

MICU1-YFP alone has no FRET signal after histamine stimulation

Average curves showing changes of the MICU1 FRET ratio of cells co-expressing the combination of MICU1-CFP and MICU1-YFP (black curve, n=19) or MICU1-YFP alone (green dotted curve, n=13) upon cell treatment with 100 µM histamine in calcium free medium. *P < 0.05 vs. MICU1-CFP and MICU1-YFP.

SUPPLEMENTARY FIGURE 2:

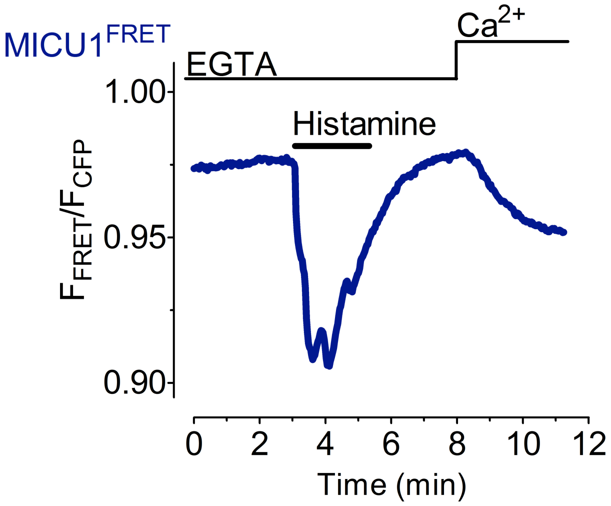

Rearrangemnet of MICU1 multimers in response to ER Ca^{2+} release and Ca^{2+} entry

Representative changes of the MICU1 FRET ratio over time in HeLa cells co-expressing MICU1-CFP and MICU1-YFP. Cells were treated with 100 µM histamine in the nominal absence of Ca^{2+}. As indicated after removal of the IP_3-generated agonist 2 mM Ca^{2+} was added.

SUPPLEMENTARY FIGURE 3:

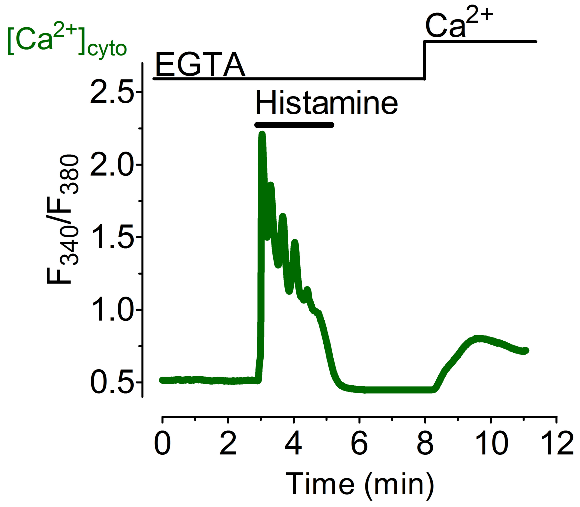

Cytosolic Ca^{2+} signals upon ER Ca^{2+} release and Ca^{2+} entry

Representative changes of $[Ca^{2+}]_{cyto}$ over time in HeLa cells that were loaded with fura-2/AM. Cells were treated with 100 µM histamine in the nominal absence of Ca^{2+}. As indicated after removal of the IP_3-generated agonist 2 mM Ca^{2+} was added.

SUPPLEMENTARY FIGURE 4:

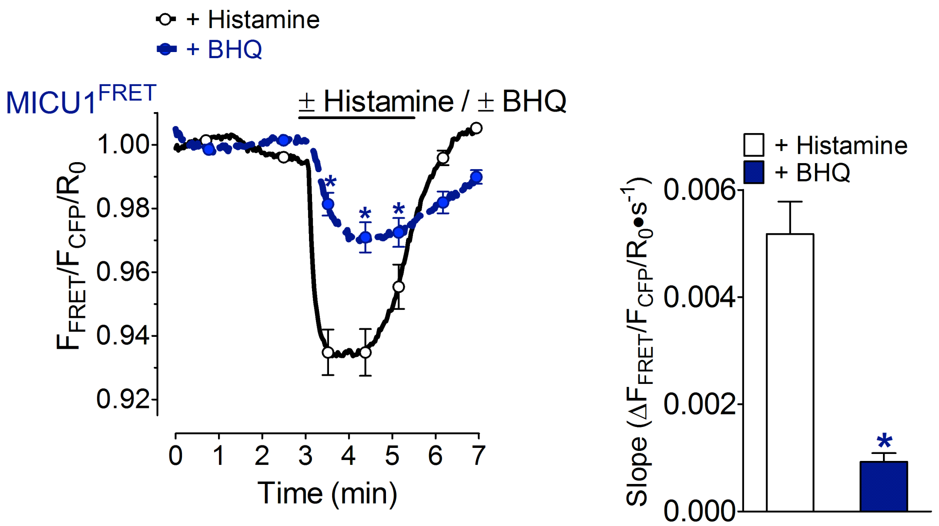

Moderate rearrangement of MICU1 multimers upon Ca^{2+} mobilization by SERCA inhibition

Left panel: Average curves showing changes of the MICU1 FRET ratio upon cell treatment with 100 µM histamine (black curve, n=19) or 15 µM BHQ (blue dotted curve, n=21). *Right panel:* Bars illustrating maximal slopes of the changes in the MICU1 FRET ratio signals (mean±SEM) in response to 100 µM histamine (white column, n=19) or 15 µM BHQ (blue filled column, n=21). Cells were treated with histamine or BHQ in Ca^{2+}- free medium. *P < 0.05 vs. Histamine.

SUPPLEMENTARY FIGURE 5:

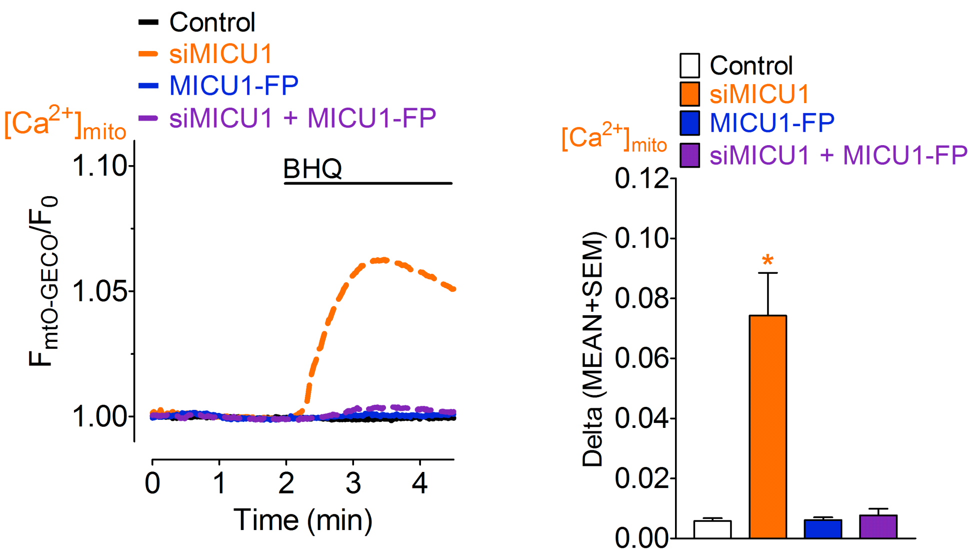

Expression of MICU1-CFP and/or -YFP rescues siRNA-mediated knock-down of MICU1

Average data and statistics of mitochondrial Ca^{2+} uptake in response to BHQ in control cells (black curve and white column, n=27), cells treated with the 3´UTR-siRNA against MICU1 (orange dotted curve and orange column, n=35), overexpressing C-terminal FP-tagged MICU1 (blue curve and blue column, n=20) or transfected with a combination of siRNA against MICU1 and FP-tagged MICU1 (red dotted curve and red column, n=20). HeLa cells expressing mtO-GECO1 were treated with 15 µM BHQ in the absence of extracellular Ca^{2+}. *$P < 0.05$ vs. Control.

SUPPLEMENTARY FIGURE 6:

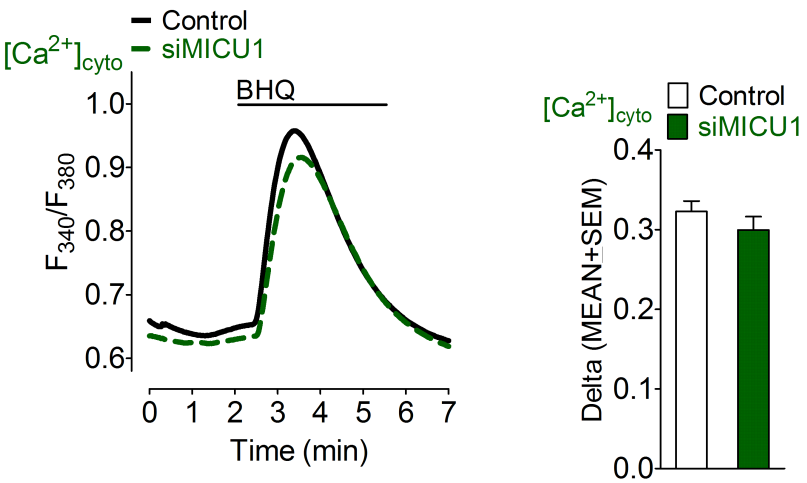

siRNA-mediated knock-down of MICU1 does not significantly affect cytosolic Ca^{2+} signals upon Ca^{2+} mobilization by the SERCA inhibitor BHQ

Average data and statistics of cytosolic Ca^{2+} signals. HeLa cells were stimulated with 15 µM BHQ in Ca^{2+}-free medium under control condition (black curve, white column, n=13) and in cells reduced of MICU1 (siRNA, green dotted curve and green column, n=14).

SUPPLEMENTARY FIGURE 7:

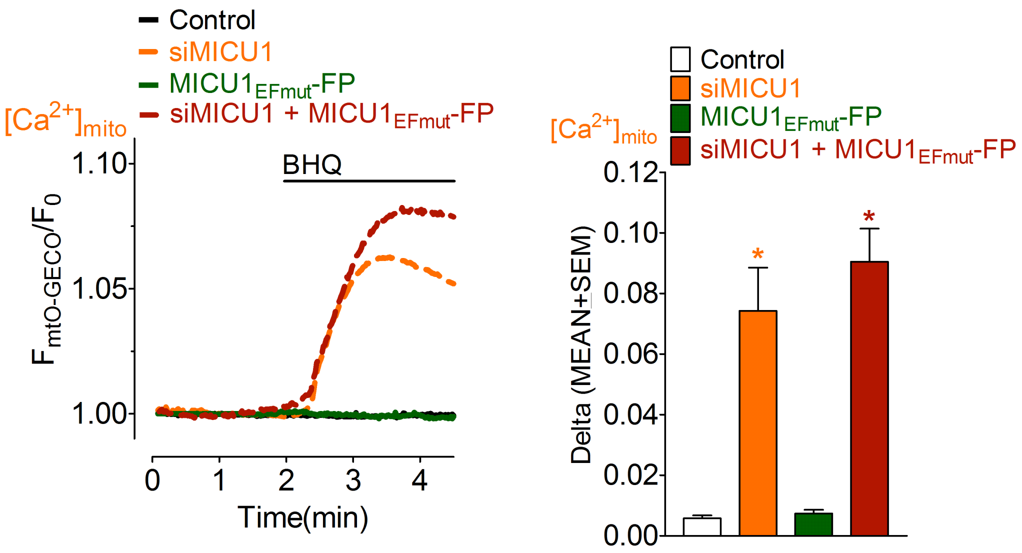

Expression of EF hand mutated MICU1-CFP and/or -YFP is unable to rescue siRNA-mediated knock-down of MICU1

Average data and statistics of mitochondrial Ca^{2+} uptake measured by mtO-GECO1 in response to 15 µM BHQ in the absence of extracellular Ca^{2+} in control cells (black curve and white column, n=27), cells treated with siRNA against MICU1 (orange dotted curve and orange column, n=35), overexpression of EF hand mutated MICU1 C-terminally tagged with FP (green curve and green column, n=19) or transfected with a combination of siRNA against MICU1 and FP-tagged EF hand mutated MICU1 (red dotted curve and red column, n=18). *P < 0.05 vs. Control.

SUPPLEMENTARY FIGURE 8:

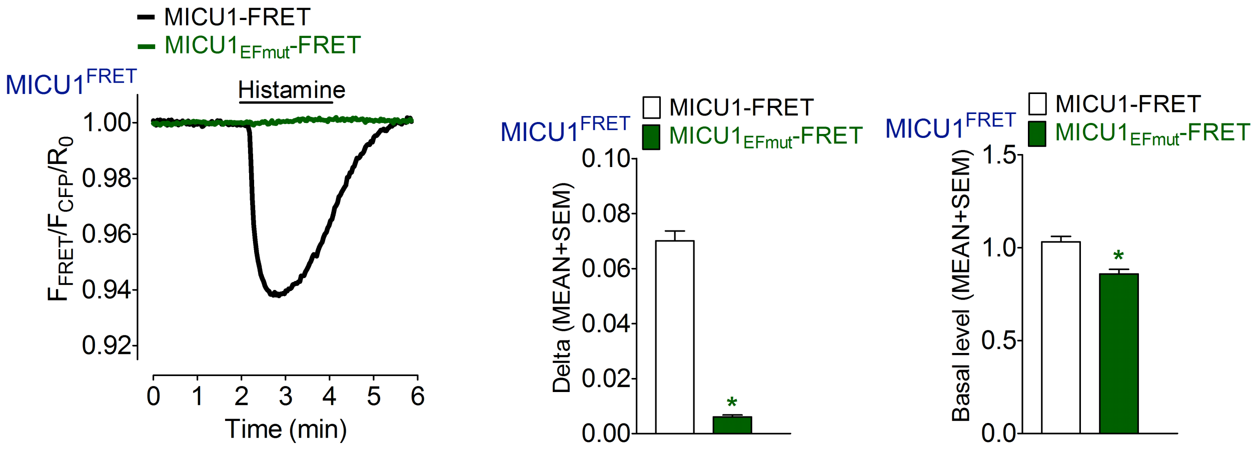

Co-expression of EF hand mutated MICU1-CFP and -YFP is unable to rearrange MICU1 FRET

Average data and statistics of MICU1 FRET in control cells (black curve and white columns, n=31) and in cells overexpressing of EF hand mutated MICU1 C-terminally tagged with CFP and YFP (green curve and green column, n=30) *P < 0.05 vs. Control.

SUPPLEMENTARY FIGURE 9:

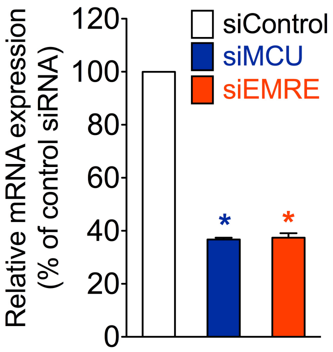

MCU and EMRE gene expression after siRNA-mediated knock-down

Bars illustrate relative mRNA expression levels (mean±SEM) of control cells (white bar, 100 %, n=4), cells treated with siRNA against MCU (blue column, n=4) and cells treated with siRNA against EMRE (red column, n=4). *P < 0.05 vs. Control.

SUPPLEMENTARY FIGURE 10:

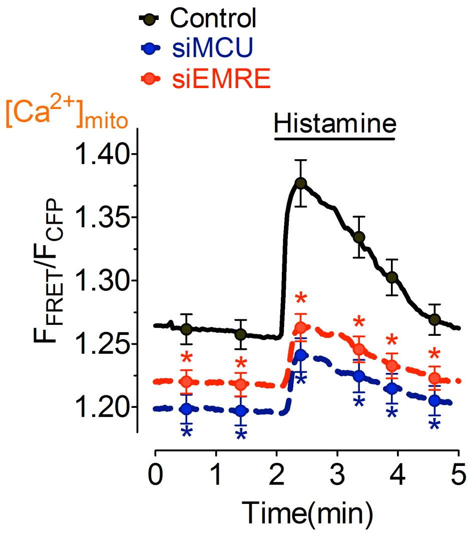

Knock-down of either MCU or EMRE strongly reduces mitochondrial Ca^{2+} uptake

Average curves showing mitochondrial Ca^{2+} signals over time in control cells (black curve, filled circles, n= 13), cells treated with siRNA against MCU (blue dotted curve, blue filled circles, n=10), and cells treated with siRNA against EMRE (red curve, red filled circles, n=15). Cells transfected with 4mtD3cpv were treated with 100 µM histamine in Ca^{2+}-free experimental buffer. mean±SEM. *P < 0.05 vs. Control.

SUPPLEMENTARY FIGURE 11:

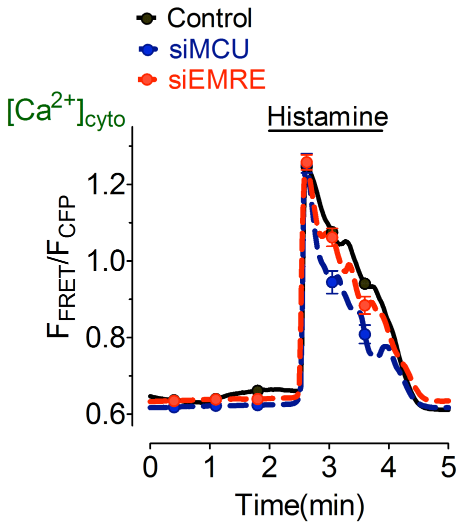

Knock-down of either MCU or EMRE has no effect on cytosolic Ca^{2+} level.

Average curves showing cytosolic Ca^{2+} level in control cells (black curve, filled circles, n=21), cells treated with siMCU (blue dotted curve, blue filled circles, n=15), and cells treated with siEMRE (red curve, red filled circles, n=18). Cells were loaded with fura-2/AM and stimulated with 100 µM histamine in Ca^{2+}-free buffer.

SUPPLEMENTARY FIGURE 12:

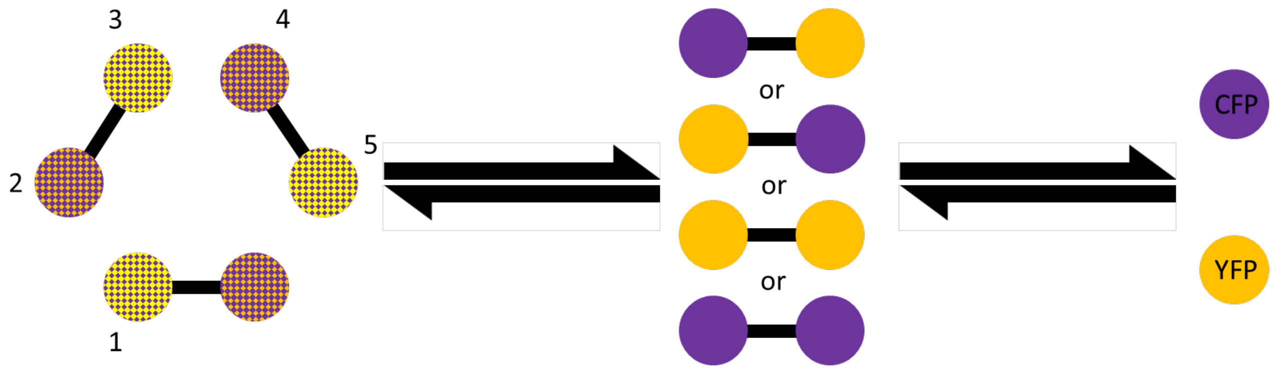

Model of the estimated arrangements of MICU1-CFP and -YFP.

On the basis of single monomers (right) MICU1 forms four different dimers (middle). These can be incorporated into hexamers (left) to 64 different variants. Numbering accounts for defining the structural orientation needed for Supplementary Equations 13, 14, and 15 in Supplementary Method.

SUPPLEMENTARY METHOD

Theoretical calculation of maximum FRET probabilities upon MICU1 rearrangement

Under the assumption that of FRET-signals are not donor limited[1], the probability of a MICU1 dimer to be a FRET-pair is determined by Supplementary Equation 1:

$$p(Dimer)_{FRET} = p(CFP/YFP) + p(YFP/CFP) \tag{1}$$

The occurrence probability of a CFP/YFP pair is determined by the expression ratio of CFP and YFP and it follows Supplementary Equation 2:

$$p(Dimer)_{FRET} = \frac{[CFP]}{[CFP]+[YFP]} \cdot \frac{[YFP]}{[CFP]+[YFP]} + \frac{[YFP]}{[CFP]+[YFP]} \cdot \frac{[CFP]}{[CFP]+[YFP]}$$

$$p(Dimer)_{FRET} = 2 \cdot \left(\frac{[CFP] \cdot [YFP]}{([CFP]+[YFP])^2} \right) \tag{2}$$

were [CFP] and [YFP] are the molar concentrations. Within a hexamer the intramolecular dimer-FRET-pair are still present (see Supplementary Equation 3):

$$p(Hexamer)_{FRET,\text{int}ra} = p(Dimer)_{FRET} \tag{3}$$

But intermolecular FRET pairs within the hexamer are added like shown in (see Supplementary Equation 4):

$$p(Hexamer)_{FRET} = p(Hexamer)_{FRET,\text{int}ra} + p(Hexamer)_{FRET,\text{int}er} \tag{4}$$

The probability of a FRET-pair between neighboring dimers is given by (see Supplementary Equation 5):

$$p(Hexamer)_{FRET,\text{int}er} = p(CFP_{1,2}) \cdot p(YFP_{2,1}) + p(YFP_{1,2}) \cdot p(CFP_{2,1}) \tag{5}$$

with $p(FP_{\text{dimer,position}})$.

The probability is determined again by the ratio of MICU1-CFP and -YFP constructs by (see Supplementary Equation 6):

$$p(Hexamer)_{FRET,\text{int}er} = \frac{[CFP]}{[CFP]+[YFP]} \cdot \frac{[YFP]}{[CFP]+[YFP]} + \frac{[YFP]}{[CFP]+[YFP]} \cdot \frac{[CFP]}{[CFP]+[YFP]} \tag{6}$$

and summarized to (see Supplementary Equation 7):

$$p(Hexamer)_{FRET,inter} = 2 \cdot \left(\frac{[CFP] \cdot [YFP]}{([CFP]+[YFP])^2} \right) \quad (7)$$

A simple approach would summarize the FRET occurrence probability to (see Supplementary Equation 8):

$$p(Hexamer)_{FRET} = 2 \cdot \left(\frac{[CFP] \cdot [YFP]}{([CFP]+[YFP])^2} \right) + 2 \cdot \left(\frac{[CFP] \cdot [YFP]}{([CFP]+[YFP])^2} \right) \quad (8)$$

and results in a halved FRET intensity after hexamer disassembly into dimers (see Supplementary Equation 9).

$$p(Hexamer)_{FRET} = 2p(Dimer)_{FRET} \quad (9)$$

Not only direct neighboring FPs (1,2) can build a intermolecular FRET-pair within a hexamer. Also FPs on position 3,4, and 5 can interact with position 1 (see Supplementary Figure 12). This results in Supplementary Equation 10:

$$\begin{aligned} p(Hexamer)_{FRET} &= p(Hexamer)_{FRET,intra} + p(Hexamer)_{FRET,inter(1,2)} \\ &+ 2 \cdot p(Hexamer)_{FRET,inter(1,(3,5))} + p(Hexamer)_{FRET,inter(1,4)} \end{aligned} \quad (10)$$

The energy transfer rate (k_{ET}) of FRET is given by Supplementary Equation 11:

$$k_{ET} = k_D \frac{R_0^6}{r^6} \quad (11)$$

were (k_D) is the donor radiation rate, (R_0) is the FRET-pair specific Förster radius, and (r) the distance between both FPs. The energy transfer rate is anti-proportional to the distance between the FPs (see Supplementary Equation 12).

$$k_{ET} \sim \frac{1}{r^6} \quad (12)$$

If we assume a perfect hexamer the intramolecular distance between FP within a dimer is equal the intermolecular distance to the neighboring FP within the hexamer. The distances between the positions are determined by (see Supplementary Equation 13-15):

$$d_{1,2} = r \quad (13)$$

$$d_{1,(3,5)} = r \cdot \sqrt{3} \tag{14}$$

$$d_{1,4} = 2 \cdot r \tag{15}$$

To include the shape of a hexamer into the model the FRET-pair probabilities are normalized as follows (see Supplementary Equation 16-18):

$$p(Hexamer)_{FRET,\text{inter}(1,2)} = p(Hexamer)_{FRET,\text{inter}} \cdot \frac{1}{(1)^6} \tag{16}$$

$$p(Hexamer)_{FRET,\text{inter}(1,2)} = p(Hexamer)_{FRET,\text{inter}} \cdot \frac{1}{(\sqrt{3})^6} \tag{17}$$

$$p(Hexamer)_{FRET,\text{inter}(1,4)} = p(Hexamer)_{FRET,\text{inter}} \cdot \frac{1}{(2)^6} \tag{18}$$

Insertion of Supplementary Equation 16-18 into Supplementary Equation 11 results in (see Supplementary Equation 19):

$$\begin{aligned} p(Dimer)_{FRET} &= 2 \cdot \left(\frac{[CFP] \cdot [YFP]}{([CFP]+[YFP])^2} \right) \cdot X \\ &= 2 \cdot \left(\frac{[CFP] \cdot [YFP]}{([CFP]+[YFP])^2} \right) + 2 \cdot \left(\frac{[CFP] \cdot [YFP]}{([CFP]+[YFP])^2} \right) \cdot \frac{1}{(1)^6} \\ &+ 4 \cdot \left(\frac{[CFP] \cdot [YFP]}{([CFP]+[YFP])^2} \right) \cdot \frac{1}{(\sqrt{3})^6} + 2 \cdot \left(\frac{[CFP] \cdot [YFP]}{([CFP]+[YFP])^2} \right) \cdot \frac{1}{(2)^6} \end{aligned} \tag{19}$$

After solving the equation;

$$X = 2 + \frac{2}{(\sqrt{3})^6} + \frac{1}{(2)^6} = 2.09$$

the $FRET_{hexamer}$ is 2.09 times higher than the $FRET_{dimer}$ (see Supplementary Equation 20):

$$p(Dimer)_{FRET} \cdot 2.09 = p(Hexamer)_{FRET} \tag{20}$$

References

1. Koushik SV, Blank PS, Vogel SS (2009) Anomalous Surplus Energy Transfer Observed with Multiple FRET Acceptors. PLoS ONE 4(11): e8031. doi: 10.1371/journal.pone.0008031
